# Variability of Total Volatile Organic Compounds (TVOC) in the Indoor Air of Retail Stores

**DOI:** 10.3390/ijerph16234622

**Published:** 2019-11-21

**Authors:** Chunrong Jia, Kevin Cao, Riya Valaulikar, Xianqiang Fu, Anna Bess Sorin

**Affiliations:** 1 School of Public Health, University of Memphis, Memphis, TN 38152, USA; xfu@memphis.edu; 2 White Station High School, Memphis, TN 38117, USA; kcao578@gmail.com; 3 St. Mary’s Episcopal School, Memphis, TN 38117, USA; rgvalaul@gmail.com (R.V.); absorin@stmarysmemphis.net (A.B.S.)

**Keywords:** retail store, volatile organic compound, VOC, TVOC, variability

## Abstract

Volatile organic compounds (VOCs) are released to the indoor air of retail stores from numerous products and activities, but available literature lacks a systematic understanding of the variability of VOC concentrations. In this study, we measured concentrations of total VOCs (TVOC) in 32 retail stores using a high-sensitivity photoionization detector (PID). Indoor thermal comfort parameters, including temperature, relative humidity, and air velocity, were simultaneously measured using an anemometer. The store-level TVOC concentrations ranged from 30 to 869 ppb and exceeded the LEED guideline in 31 stores. TVOC levels were notably high in hardware stores (median = 536 ppb, *p* = 0.0002) and paints, household, and home accessories sections within stores (*p* < 0.05). TVOC levels were elevated in mornings and evenings, possibly due to low ventilation and cleaning activities at the beginning and end of business hours. The between-store, within-store, and temporal variations accounted for 85%, 0.5%, and 14% of the total variance, respectively. The variance structure suggested that in-store VOC concentrations were predominantly driven by their source location, and representative monitoring should first consider covering various store types. Current store VOC levels present health concerns, but further studies are needed to evaluate risks among customers.

## 1. Introduction

Retail stores are an indoor environment where people spend a substantial portion of their time away from home. An early study with 2697 subjects reported that people spent 60 min/day (median) in stores [1]. The Census Bureau’s data showed that the civilian population spent 43 min/day purchasing goods and services in 2017 [2], and the time was decreasing [3]. Despite the growth of online shopping, consumers still spend significantly more time per visit in-store than online [4]. Interestingly, the purpose of shopping is not only for buying goods and services but for reducing sadness and stress [5,6]. Studies have documented the positive impacts of shopping on mood, i.e., “retail therapy” [7].

Indoor air quality (IAQ) in stores is a key topic when studying the health benefits of shopping. Among many IAQ parameters, volatile organic compounds (VOCs) are a focus as they emit from numerous products and activities, e.g., detergents, paints, solvents, tools, clothes, toys, cleaning, and cooking. VOCs are hydrocarbons that exist as gases or vapors at room temperature [8]. In the U.S., studies have reported that aldehyde, aromatic, aliphatic, halogenated, and terpenoid compounds are the VOCs commonly detected in commercial buildings [9,10,11,12]. For example, in 21 California stores, the most abundant VOCs were formaldehyde, acetaldehyde, hexanal, toluene, mixed xylenes, and acetone, with mean concentrations at >10 μg/m^3^ [12]. A store study in Boston, Massachusetts, reached similar findings, i.e., formaldehyde, acetaldehyde, toluene, and mixed xylenes were the predominant VOCs with geometric means over 10 μg/m^3^ [9]. Indoor VOC concentrations are typically 5–10 times higher than outdoor levels [13]. A review of store IAQ studies shows that half of the stores do not have sufficient ventilation, which may cause trapping of indoor air pollutants [14]. Research has found that stores have the highest indoor VOC levels compared to other common microenvironments like residences, offices, or even vehicles [9,15]. Airborne VOCs even at low concentrations are known to cause a series of respiratory illnesses [16,17], and studies have reported sick building symptoms (SBS) and impaired productivities among store workers [18,19]. Improving store IAQ not only protects human health but promotes business [20].

Understanding what factors determine indoor VOCs is a critical step toward evaluating health risks and designing control strategies. Data are insufficient regarding VOC levels and their influencing factors in stores, setting a barrier to adequate controls and regulations [14]. A few case studies provide detailed analyses of VOC variability in individual large shopping malls [21,22], but no study has been specifically designed to examine how indoor VOC levels vary between stores and spatially within stores. This study was designed to fill this knowledge gap by measuring concentrations of total VOCs (TVOC) in different categories of stores. We then partitioned the total variance to between-store, within-store, and temporal variations, and conducted a screening-level risk assessment.

## 2. Methods

### 2.1. Field Sampling Design

The investigators randomly selected 32 different retail stores in the Greater Memphis Area, Tennessee, U.S.A., in summer 2019. These stores included 6 supermarkets, 6 grocery stores, 8 hardware stores, 6 specialty stores, and 6 multipurpose stores. All the stores were freestanding, big-box style stores. The summer was chosen for the convenience and availability of field staff. The field sampling was conducted in two stages. First, TVOC was measured three times (7:00 a.m.–10:00 a.m., 2:00 p.m.–4:00 p.m., and 7:30 p.m.–9:00 p.m.) in five stores to examine between-store, within-store, and temporal variability. The five stores each represented a store type. In the second stage, TVOC was measured cross-sectionally in an additional 27 different stores. The field investigators visited these stores at random time points between 10:00 a.m. to 9:00 p.m. During each store visit, outdoor TVOC was first measured at a location outside of the store. Then, indoor TVOC was measured at multiple sections, including home accessories, household, paints, self-care, and food. The entire field sampling included 42 unique store visits.

### 2.2. Measurement Methods for Environmental Parameters

Multiple environmental quality parameters were measured at each sampling location. TVOC was measured using a photoionization detector (PID, Model: ppbRAE3000, RAE Systems, Inc., Sunnyvale, CA, USA). The PID has been demonstrated as a rapid, effective instrumental technique to evaluate indoor VOCs and human VOC exposures [23,24,25]. The PID in this study was equipped with a 10.6 eV lamp that had a resolution of 1 ppb in the range of 0–9999 ppb. In the field, it ran for at least 3 min at each location at a data recording frequency of one reading per 5 s. This sampling protocol yielded at least 30 data points, and the average represented the TVOC concentration for that section. Simultaneously, thermal comfort parameters, including temperature, relative humidity (RH), and air velocity, were measured using a hygro-anemometer (Model: HHC261, Omega Engineering, Inc., Norwalk, CT, USA). Their average readings were recorded directly from the instrument’s LCD panel. All the instruments were placed on a shopping cart at the center of each section and ran for at least 3 min. To ensure data quality, the PID was calibrated weekly following the manufacturer’s manual. The calibration was performed at two points: the zero calibration was determined with a zero (VOC-free) air and the span calibration with a 10-ppm isobutylene standard gas. The uncertainty was ±3% at the 10-ppm calibration point. The instruments were equilibrated for at least 5 min after they were brought from outside to inside. The PID featured built-in humidity compensation with integral humidity and temperature sensors, and thus automatically corrected TVOC readings for RH [26].

### 2.3. Data Analysis

We calculated descriptive statistics (including central tendency, variation, and percentiles) for all environmental parameters based on store-level measurements (n = 32). The store-level measurement of a parameter (e.g., TVOC) was the average of all measurements taken at each of the five sections. For stores with three visits, we first averaged multi-time measurements for each section and then averaged these five section-level measurements to obtain the store-level measurement. Concentrations of TVOC in each store and section generally displayed log-normal distributions, and thus the data were log-transformed for the following statistical analyses. The total variance of indoor TVOC concentrations was apportioned to between-store, within-store, and temporal variations using Proc Nested in SAS (Ver 9.4, SAS Institute Inc., Cary, NC, USA). The rationale and applications of variance analysis for understanding the variance structure of indoor VOCs have been described previously [27]. Comparisons of TVOC levels by store type, section type, and monitoring time were performed using linear mixed effect models. Rank-based Spearman correlation was used to measure the association between indoor TVOC and other parameters. Except for the nested analysis, all the analyses were performed in R (Ver. 3.6.1).

The health risk of indoor TVOC exposure was evaluated by comparing the TVOC concentrations with the guideline value. Currently, there is no health standard for indoor TVOC levels in non-industrial settings [28,29]. We thus adopted the guideline of 500 μg/m^3^ set by the Leadership in Energy and Environmental Design (LEED), the most widely used green building rating system [30]. The use of LEED criteria to evaluate TVOC risks has been a common practice in previous building studies [31]. The 500 μg/m^3^ is a toluene-equivalent concentration measured using charcoal sampling followed by gas chromatography (GC) analysis and a flame ionization detector (FID) or a mass spectrometer (MS). It is equivalent to 108 ppb isobutylene-based PID concentration [26], following the conversion method described in Appendix A.

## 3. Results

### 3.1. Indoor TVOC Levels and the Health Risks

The grand average TVOC concentration was 271 ppb, with a coefficient of variance of 71% in the 32 stores (Table 1). The lowest concentration of 30 ppb occurred in a supermarket and the highest concentration of 869 ppb in a hardware store. Store-level TVOC concentrations exceeded the LEED guideline of 108 ppb in all stores except a supermarket. Out of the total of 214 sections investigated, 175 sections (81%) had TVOC concentrations exceeding 108 ppb. A closer look revealed that the exceedance occurred in 92%, 94%, and 96% of household, home accessories, and paints sections, respectively, and 63% and 65% of self-care and food sections, respectively.

Temperature and RH were stable inside stores: temperature ranged from 24 to 26 °C and RH from 38% to 57% in most stores (Table 1). Indoor airflow was mostly undetectable. These ventilation parameters showed no statistical differences among the five types of stores (*p* > 0.2). For indoor thermal comforts, the American Society of Heating, Refrigerating and Air-Conditioning Engineers (ASHRAE) requires that temperature be 19–28 °C, RH 25%–60%, and air velocity less than 0.25 m/s during the cooling mode [32]. The indoor comfort parameter measurements suggest store ventilation systems in all the stores were designed and operated to meet the ASHRAE standards [32,33].

### 3.2. Variability of Store TVOC Concentrations

The nested analysis results showed that between-store, within-store spatial, and temporal variations represented 85%, 0.5%, and 14% of the total variation of indoor TVOC concentrations, respectively. This variance structure indicated that VOC concentrations differed significantly among stores (*p* < 0.001), and had some temporal variation (*p* = 0.062). The spatial variation of TVOC concentrations within stores was almost negligible in comparison to between-store and temporal variations.

**Figure 1:**
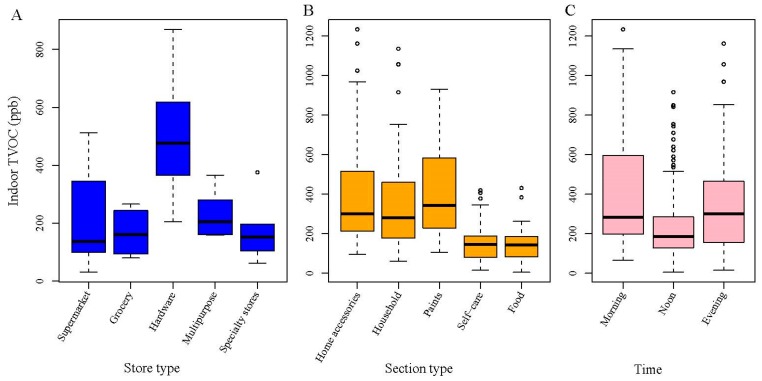
Comparison of indoor total volatile organic compounds (TVOC) concentrations by store type, store section type, and time. (**A**) Comparison by store type; (**B**) Comparison by section type; and (**C**) Comparison by time. The midline of a box is the median of the data, and the upper and lower limits are the third and first quartiles, respectively. The whiskers extend 1.5 times the interquartile range from the box, and dots represent individual data points as outliers.

By store type, the median store-level TVOC concentrations were 141, 152, 536, 235, and 151 ppb in supermarket, grocery, hardware, multipurpose, and specialty stores, respectively (Figure 1A). Hardware stores had significantly higher TVOC concentrations than other stores (*p* = 0.0002), and the other types of stores had similar TVOC concentrations (*p* = 0.12).

Regarding the temporal variation, our limited data showed that TVOC concentrations were the lowest in the afternoons (*p* < 0.01, Figure 1C). The higher concentrations in mornings and evenings might have resulted from lower ventilation during non-business hours at night for the energy-saving purpose.

TVOC concentrations also displayed some within-store variation. From Figure 1B, store sections could be classified into high- and low-VOC sections: the former included home accessories, household, and paints sections, and the latter included self-care and food sections. The TVOC concentrations differed significantly between two groups (*p* < 0.05) but were similar within each group (*p* > 0.1).

The Spearman correlations of indoor TVOC concentration were all insignificant (*p* > 0.05) with outdoor TVOC concentration, outdoor temperature, outdoor RH, indoor temperature, indoor RH, or indoor air velocity. The only exception was the negative association between indoor TVOC level and outdoor air velocity (Spearman r = −0.346, *p* = 0.027); however, we would consider it no association as this coefficient was small and lacked a solid scientific explanation.

## 4. Discussion

Few studies have measured TVOC in various microenvironments in the U.S. The sum of all target VOCs had mean values of 115, 233, and 106 μg/m^3^ in grocery, furniture/hardware, and apparel stores, respectively, in California, USA [12]; however, these concentrations only represented a fraction of the TVOC. For residential indoor air, a study reported an average (±SD) TVOC concentration of 327 (±119) ppb in 58 homes in Niagara Falls City, NY [34]. In 37 US schools, the total target VOC (TTVOC) concentration averaged 42 μg/m^3^ with a maximum of 197 μg/m^3^ [35]. By summarizing the Building Assessment Survey and Evaluation (BASE) Study data, Rackes and Waring reported that the median TVOC concentration was 260.5 μg/m^3^ in offices in six major US cities [36]. In vehicles, TVOC levels were 400–800 μg/m^3^ at 80 °F but could be five-fold higher under extreme heat conditions [37]. Extremely high TVOC concentrations occur in some occupational settings, e.g., TVOC concentration averaged 12 ppm (range 0.035–67 ppm) in New York City nail salons [38]. Although not always directly comparable due to different measurement techniques, the TVOC levels in this study were generally higher than those detected in other non-occupational indoor environments. Studies of individual VOCs in multiple settings also reached similar results [9,15].

Our analyses showed that indoor TVOC concentrations varied mostly by store type and were not associated with other environmental parameters. In Boston, the highest toluene and formaldehyde concentrations were detected in multipurpose and housewares stores, respectively [9]. In Texas and Pennsylvania, two grocery stores had the highest levels of ethanol and acetaldehyde, possibly resulting from baking activities [39]. In California, high VOC concentrations in certain building categories could be linked to expected sources [10]. These facts suggest that indoor sources are the predominant determinant of indoor TVOC in retail stores.

Ventilation is assumed as the best approach to improve IAQ when source control is not an option. ASHRAE has established Standard 62.1 that requires minimum ventilation rates in commercial buildings to preserve “occupants’ health, safety and well-being” [29]; however, a critical review found that half of the stores failed to meet relevant ventilation standards [14]. Also, previous studies reported inconsistency in IAQ improvement by increased ventilation: some were effective [40], and some were not [14,41]. Concentrations of fine particulate matter, formaldehyde, and acetaldehyde in stores often exceeded the guidelines, despite stores’ compliance with ASHRAE Standard 62.1 [14]. A study by the Lawrence Berkeley National Laboratory concludes that increasing ventilation to improve IAQ may not be effective, nor is it economical [42]. In this monitoring campaign, we observed higher TVOC concentrations in mornings and evenings, possibly due to reduced ventilation operations at night [43,44] or the cleaning activities that occurred before stores opened and after they were closed [21]. It was unfortunate that we could not measure ventilation rates, which restricted us from further studying the association between ventilation and in-store TVOC. 

This study yielded a variance structure that could guide future store IAQ monitoring. The large between-store variation (85%) indicates that obtaining representative store IAQ data requires selecting a wide variety of store types. When collecting samples within a store, it is more important to collect samples at multiple time points or to collect time-integrated samples. The small within-store spatial variation (only 0.5%) indicated well-mixed air conditions in these stores, which was also reported in the authors’ previous study of large commercial buildings [11]. Thus, the need to use multiple sampling locations within a store is minimal, given the spatial uniformity of VOC concentrations. However, this rule may only apply to big-box stores in this study. In non-freestanding shopping malls, the complex ventilation methods, airflows, and building structures resulted in tremendous heterogeneity in indoor air pollutants, warranting multi-point sampling [21,22,45].

Reaching a definite answer to the health impacts of store VOC contamination is still challenging. Acute effects are not expected among customers as they typically spend a short time in stores, and store TVOC levels are still far below the acute effect thresholds. Previous store studies also reported that individual VOC levels were lower than the health criteria [10,39]. Health concerns may arise for vulnerable sub-populations (e.g., children) and store staff. A population-based Australian study reported that children exposed to TVOC of >60 μg/m^3^ had a fourfold increased risk of having asthma [46]. A school IAQ study in Porto, Portugal, showed that high levels of TVOC were associated with higher odds of wheezing in children [47]. Similarly, studies have reported health symptoms and lowered productivity among store staff. In Seoul, Korea, half of 314 store workers in nine shopping centers experienced SBS in their work [18]. In West China, store staff reported noticeable SBS and neutral satisfaction with store IAQ [45]. However, no study has investigated customers’ perceptions or complaints of IAQ in stores, representing a knowledge gap that needs future investigations.

We acknowledge several limitations of this study. First, the grab sampling approach could not capture the full temporal variation of indoor air pollution. On the one hand, a representative sample always requires continuous or integrate sampling for an extended period; on the other hand, a 3-min sampling duration could simulate the typical duration a customer spends at a specific store section. The total of over 200 samples in this study could capture customers’ exposures at different locations in various stores, and thus, to a large extent, represented customers’ VOC exposures during shopping. Second, we monitored IAQ in stores just in summer, which might represent the worst-case according to the findings in a previous multi-season multi-store study (Loh et al., 2006). Third, we only investigated the big-box stores, which are the most common commercial building type in this region. Our findings may not apply to those large-area multi-story and multi-compartment shopping malls [21,22,45]. Fourth, we generally followed the Harvard study [9] to categorize store types, but the classifications might not be exact or definite due to complicated situations in each store. Fifth, the PID, as a non-specific measurement technique, could not reflect the complex VOC compositions. The findings of TVOC variability might not be extended to individual VOC components, warranting the need to characterize specific VOCs. In response to this need, new instrumental developments have been attempted to take advantage of low-cost portable PID sensors while overcoming the non-specificity, e.g., GC-PID [48] and GC × GC-PID [49]. Finally, there are no health criteria for indoor TVOC, and the definitions of TVOC are inconsistent [50].

The handheld PID monitor has several advantages in comparison to the gold-standard GC-MS analysis for IAQ investigations. The cost of equipment purchase (a few thousand US dollars) and operations (almost 0) are much lower for PIDs, and new low-cost PID sensors are emerging in the market [51]. A PID provides rapid direct measurements of TVOC so that IAQ problems can be quickly identified, evaluated, and remediated. In contrast, it is costly (over $100/sample) and time-consuming (days or weeks) to complete field VOC sample collection and laboratory GC/MS analyses. In such circumstances, IAQ problems may not be solved timely and can grow into a major incident. Our study added a new application of the PID technique for characterizing indoor pollution and improving source control, as suggested by an early review [50].

## 5. Conclusions

This study observed the highest TVOC concentrations in hardware stores, and in paints, home accessories, and household sections within stores. A systematic variance analysis showed that indoor TVOC levels were predominantly driven by sources, suggesting the most effective approach to improve IAQ is by modifying or limiting emissions from store-related sources. TVOC levels exceeded the LEED guideline in 97% of stores, presenting health concerns for store workers as well as customers. We have recognized the following three future research needs: (1) measure long-term exposures to specific VOCs in a wide variety of stores; (2) conduct environmental epidemiologic studies targeting retail store shoppers; and (3) establish TVOC guideline values based on the PID measurement technique.

## Figures and Tables

**Table 1:** Descriptive statistics of indoor environmental parameters measured in 32 retail stores.

Parameter	Unit	Mean	SD	GM	GSD	Min	Median	P90	Max
Indoor TVOC	ppb	271	194	212	2	30	202	510	869
Indoor temperature	°C	24.8	1.3	24.7	1.1	22.2	25.0	25.9	27.6
Indoor humidity	%	45.7	7.5	45.2	1.2	34.1	43.6	57.4	64.4
Indoor air velocity	m/s	0.0	0.1	NA	NA	0.0	0.0	0.1	0.2
Outdoor TVOC	ppb	22	22	NA	NA	0	19	44	78
Outdoor temperature	°C	29.5	3.2	29.3	1.1	20.2	30.0	33.3	34.8
Outdoor humidity	%	48.9	16.5	46.0	1.5	22.2	52.0	66.1	79.1
Outdoor air velocity	m/s	1.0	0.7	0.8	2.15	0.2	1.0	2.2	3.0

Notes: SD—Standard deviation; GM—Geometric mean; GSD—Geometric standard deviation; Min—Minimum; P90—the 90th percentile; Max—Maximum.

## Appendix A

Conversion from a GC-based TVOC concentration to a PID-based TVOC concentration:

The PID is calibrated with 10-ppm isobutylene (IBE) standard gas, and thus the readings are IBE-based concentrations. For a common VOC mixture, there is a close relationship between the TVOC measurement by a PID and that by charcoal sampling followed by GC-FID analysis (R^2^ = 0.95) [25]:

ln C_PID_ = 0.04 + 0.95 ln C_GC_, (A1)

where C_PID_ (in ppb) is the IBE-based concentration measured by PID and C_GC_ (in ppb) is the concentration determined by GC. Assuming C_GC_ is a toluene-equivalent concentration, C_GC_ in μg/m^3^ can be converted to C_GC_ in ppb using the ideal gas law:

C_GC_ (in ppb) = 24.45/92.14 × C_GC_ (in μg/m^3^), (A2)

where 24.45 is the volume (in L) of a mole (gram molecular weight) of a gas at 1 atmospheric pressure and 25 °C, and 92.14 is the molecular weight of toluene. A C_GC_ of 500 μg/m^3^ is equivalent to IBE-based PID concentration of 108 ppb, based on Equations (A1) and (A2).

## References

[1] U.S. EPA (2011). Exposure Factors Handbook, 2011 Edition, Table 16–18.

[2] U.S. Department of Labor American Time Use Survey, Table A-1. https://www.bls.gov/tus/tables.htm.

[3] Kopf D. Time Spent Shopping is Declining in the US. https://www.theatlas.com/charts/Hqt5l_oLY.

[4] First Insight I. The State of Consumer Spending: In-Store Impulse Shopping Stands the Test of Time. https://www.firstinsight.com/white-papers-posts/the-state-of-consumer-spending-report.

[5] Rick S.I., Pereira B., Burson K.A. (2014). The benefits of retail therapy: Making purchase decisions reduces residual sadness. J. Consum. Psychol..

[6] Clicktale Stress Shopping: The Relationship between Stress and Shopping in the Age of Digital Experiences. https://www.clicktale.com/media/4457/clicktale_stressshopping.pdf.

[7] Atalay A.S., Meloy M.G. (2011). Retail therapy: A strategic effort to improve mood. Psychol. Mark..

[8] U.S. EPA (1999). Compendium Method TO-15, Determination of Volatile Organic Compounds (VOCs) in Air Collected in Specially-Prepared Canisters and Analyzed by Gas Chromatograpy/Mass Spectrometry (GC/MS).

[9] Loh M.M., Houseman E.A., Gray G.M., Levy J.I., Spengler J.D., Bennett D.H. (2006). Measured concentrations of VOCs in several non-residential microenvironments in the United States. Environ. Sci. Technol..

[10] Wu X.M., Apte M.G., Maddalena R., Bennett D.H. (2011). Volatile organic compounds in small- and medium-sized commercial buildings in California. Environ. Sci. Technol..

[11] Jia C., Batterman S., Godwin C., Charles S., Chin J.Y. (2010). Sources and migration of volatile organic compounds in mixed-use buildings. Indoor Air.

[12] Chan W.R., Cohn S., Sidheswaran M., Sullivan D.P., Fisk W.J. (2015). Contaminant levels, source strengths, and ventilation rates in California retail stores. Indoor Air.

[13] Jia C., Batterman S., Godwin C. (2008). VOCs in industrial, urban and suburban neighborhoods, Part 1: Indoor and outdoor concentrations, variation, and risk drivers. Atmos. Environ..

[14] Zaatari M., Nirlo E., Jareemit D., Crain N., Srebric J., Siegel J. (2014). Ventilation and indoor air quality in retail stores: A critical review (RP-1596). HVAC R Res..

[15] Sexton K., Mongin S.J., Adgate J.L., Pratt G.C., Ramachandran G., Stock T.H., Morandi M.T. (2007). Estimating volatile organic compound concentrations in selected microenvironments using time-activity and personal exposure data. J. Toxicol. Environ. Health Part A Current Issues.

[16] Jones A.P. (1999). Indoor air quality and health. Atmos. Environ..

[17] Mendell M.J. (2007). Indoor residential chemical emissions as risk factors for respiratory and allergic effects in children: A review. Indoor Air.

[18] Kim J., Jang M., Choi K., Kim K. (2019). Perception of indoor air quality (IAQ) by workers in underground shopping centers in relation to sick-building syndrome (SBS) and store type: A cross-sectional study in Korea. BMC Public Health.

[19] Mujan I., Andelkovic A.S., Muncan V., Kljajic M., Ruzic D. (2019). Influence of indoor environmental quality on human health and productivity—A review. J. Clean Prod..

[20] WorldGBC (2016). Health, Wellbeing & Productivity in Retail: The Impact of Green Buildings on People and Profit.

[21] Amodio M., Dambruoso P.R., de Gennaro G., de Gennaro L., Loiotile A.D., Marzocca A., Stasi F., Trizio L., Tutino M. (2014). Indoor air quality (IAQ) assessment in a multistorey shopping mall by high-spatial-resolution monitoring of volatile organic compounds (VOC). Environ. Sci. Pollut. Res..

[22] Eklund B.M., Burkes S., Morris P., Mosconi L. (2008). Spatial and temporal variability in VOC levels within a commercial retail building. Indoor Air.

[23] Bocos-Bintintan V., Smolenschi A., Ratiu I.A. (2016). Rapid determination of indoor air contaminants in shoe shops using photoionization detectors. Studia Univ. Babes Bolyai Chem..

[24] Mizukoshi A., Kumagai K., Yamamoto N., Noguchi M., Yoshiuchi K., Kumano H., Yanagisawa Y. (2010). A novel methodology to evaluate health impacts caused by VOC exposures using real-time VOC and Holter monitors. Int. J. Environ. Res. Public Health.

[25] Coy J.D., Bigelow P.L., Buchan R.M., Tessari J.D., Parnell J.O. (2000). Field evaluation of a portable photoionization detector for assessing exposure to solvent mixtures. AIHAJ A J. Sci. Occup. Environ. Health Saf..

[26] RAE Systems Inc. (2013). The PID Handbook: Theory and Applications of Direct-Reading Photoionization Detectors.

[27] Jia C., Batterman S.A., Relyea G.E. (2012). Variability of indoor and outdoor VOC measurements: An analysis using variance components. Environ. Pollut..

[28] U.S. EPA Volatile Organic Compounds’ Impact on Indoor Air Quality. https://www.epa.gov/indoor-air-quality-iaq/volatile-organic-compounds-impact-indoor-air-quality.

[29] Persily A. (2015). Challenges in developing ventilation and indoor air quality standards: The story of ASHRAE Standard 62. Build. Environ..

[30] LEED (2019). LEED v4 for Building Design and Construction.

[31] Stamatelopoulou A., Asimakopoulos D.N., Maggos T. (2019). Effects of PM, TVOCs and comfort parameters on indoor air quality of residences with young children. Build. Environ.

[32] ASHRAE (2017). Standard 55-2017 Standard 55: Thermal Environmental Conditions for Human Occupancy.

[33] ASHRAE (2016). ANSI/ASHRAE Standard 62.1-2016 Ventilation for Acceptable Indoor Air Quality.

[34] Grenga P.N., Gallagher M.J., McGahan M.E., Raymond D.M., Priefer R. (2011). Assessment of airborne total volatile organic compounds of Niagara Falls residences as compared to resident lifestyle. Indoor Built Environ..

[35] Zhong L.X., Su F.C., Batterman S. (2017). Volatile organic compounds (VOCs) in conventional and high performance school buildings in the US. Int. J. Environ. Res. Public Health.

[36] Rackes A., Waring M.S. (2013). Modeling impacts of dynamic ventilation strategies on indoor air quality of offices in six US cities. Build. Environ..

[37] Fedoruk M.J., Kerger B.D. (2003). Measurement of volatile organic compounds inside automobiles. J. Expo. Anal. Environ. Epidemiol..

[38] Pavilonis B., Roelofs C., Blair C. (2018). Assessing indoor air quality in New York City nail salons. J. Occup. Environ. Hyg..

[39] Nirlo E.L., Crain N., Corsi R.L., Siegel J.A. (2014). Volatile organic compounds in fourteen U.S. retail stores. Indoor Air.

[40] Vornanen-Winqvist C., Salonen H., Jarvi K., Andersson M.A., Mikkola R., Marik T., Kredics L., Kurnitski J. (2018). Effects of ventilation improvement on measured and perceived indoor air quality in a school building with a hybrid ventilation system. Int. J. Environ. Res. Public Health.

[41] Dutton S.M., Fisk W.J. (2014). Energy and indoor air quality implications of alternative minimum ventilation rates in California offices. Build. Environ..

[42] Chan W.R., Parthasarathy S., Fisk W.J., McKone T.E. (2016). Estimated effect of ventilation and filtration on chronic health risks in US offices, schools, and retail stores. Indoor Air.

[43] Challoner A., Gill L. (2014). Indoor/outdoor air pollution relationships in ten commercial buildings: PM_2.5_ and NO_2_. Build. Environ.

[44] Zaatari M., Novoselac A., Siegel J. (2016). Impact of ventilation and filtration strategies on energy consumption and exposures in retail stores. Build. Environ..

[45] Shang Y.Z., Li B.Z., Baldwin A.N., Ding Y., Yu W., Cheng L. (2016). Investigation of indoor air quality in shopping malls during summer in Western China using subjective survey and field measurement. Build. Environ..

[46] Rumchev K., Spickett J., Bulsara M., Phillips M., Stick S. (2004). Association of domestic exposure to volatile organic compounds with asthma in young children. Thorax.

[47] Madureira J., Paciencia I., Rufo J., Ramos E., Barros H., Teixeira J.P., Fernandes E.D. (2015). Indoor air quality in schools and its relationship with children’s respiratory symptoms. Atmos. Environ..

[48] Nasreddine R., Person V., Serra C.A., Le Calvé S. (2016). Development of a novel portable miniaturized GC for near real-time low level detection of BTEX. Sens. Actuators B Chem..

[49] Pang X., Nan H., Zhong J., Ye D., Shaw M.D., Lewis A.C. (2019). Low-cost photoionization sensors as detectors in GC × GC systems designed for ambient VOC measurements. Sci. Total Environ..

[50] Molhave L., Clausen G., Berglund B., de Ceaurriz J., Kettrup A., Lindvall T., Maroni M., Pickering A.C., Risse U., Rothweiler H. (1997). Total volatile organic compounds (TVOC) in indoor air quality investigations. Indoor Air.

[51] Spinelle L., Gerboles M., Kok G., Persijn S., Sauerwald T. (2017). Review of portable and low-cost sensors for the ambient air monitoring of benzene and other volatile organic compounds. Sensors.

